# Worms and bugs of the gut: the search for diagnostic signatures using barcoding, and metagenomics–metabolomics

**DOI:** 10.1186/s13071-022-05225-7

**Published:** 2022-04-01

**Authors:** Marina Papaiakovou, D. Timothy J. Littlewood, Stephen R. Doyle, Robin B. Gasser, Cinzia Cantacessi

**Affiliations:** 1 Department of Veterinary Medicine, University of Cambridge, Cambridge, CB3 0ES UK; 2 Department of Life Sciences, Natural History Museum, Cromwell Road, London, SW7 5BD UK; 3 Wellcome Sanger Institute, Hinxton, Cambridge, CB10 1SA UK; 4 Melbourne Veterinary School, The University of Melbourne, Parkville, VIC 3010 Australia

## Abstract

Gastrointestinal (GI) helminth infections cause significant morbidity in both humans and animals worldwide. Specific and sensitive diagnosis is central to the surveillance of such infections and to determine the effectiveness of treatment strategies used to control them. In this article, we: (i) assess the strengths and limitations of existing methods applied to the diagnosis of GI helminth infections of humans and livestock; (ii) examine high-throughput sequencing approaches, such as targeted molecular barcoding and shotgun sequencing, as tools to define the taxonomic composition of helminth infections; and (iii) discuss the current understanding of the interactions between helminths and microbiota in the host gut. Stool-based diagnostics are likely to serve as an important tool well into the future; improved diagnostics of helminths and their environment in the gut may assist the identification of biomarkers with the potential to define the health/disease status of individuals and populations, and to identify existing or emerging anthelmintic resistance.

**Graphical Abstract:**

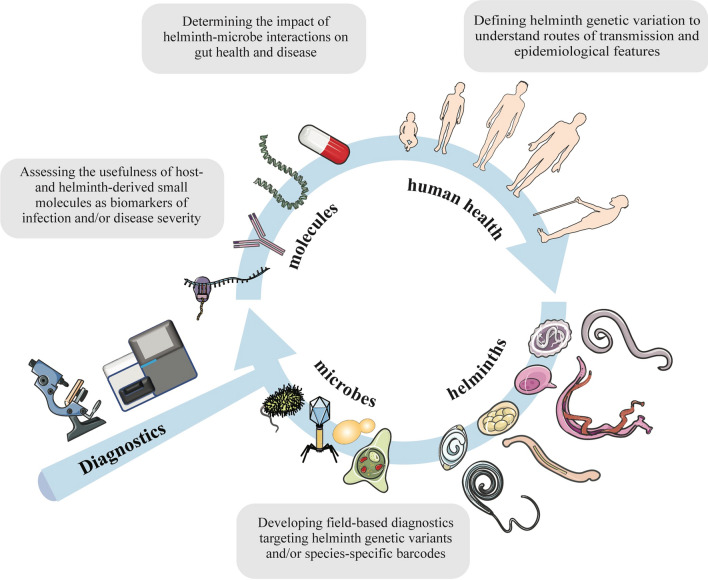

## Background

Gastrointestinal (GI) helminths cause significant disease in both humans and animals. In humans, soil-transmitted helminths (STHs; including *Ascaris lumbricoides*, *Trichuris trichiura*, *Necator americanus*, *Ancylostoma duodenale*,* Ancylostoma ceylanicum* and *Strongyloides stercoralis*), blood flukes (*Schistosoma* spp.) and food-borne liver flukes (e.g. *Clonorchis* and *Opisthorchis* spp.) afflict > 1.5 billion people globally, most of whom live in disadvantaged and neglected communities [1–4]. Children are particularly vulnerable to the clinical consequences of STH infections, which include stunted growth, malnutrition and/or anaemia [5], whilst fluke infections can ultimately lead to GI, hepatosplenic or urogenital diseases (*Schistosoma* spp.) [1] and/or cancers (*Schistosoma haematobium*, *Clonorchis sinensis* and *Opisthorchis viverrini*) [6]. These helminths contribute to a tangible reduction in quality of life, equating to > 5 million disability-adjusted life years (DALYs) for STH-infected persons [4] and > 3 million DALYs for adults infected with schistosomiasis [7]. In addition, species of GI helminths of livestock represent a major threat to food security and animal health. In Europe alone, the cost of infections by GI helminths (including the ‘barber’s pole worm’ *Haemonchus contortus*, the ‘brown stomach worm’ *Teladorsagia circumcincta* and the ‘liver fluke’ *Fasciola hepatica*) has been estimated at €1.8 billion due to production losses, the cost of treatment programmes and death [8].

Traditionally, the fight against parasitic helminths of both human and animal health importance has almost exclusively relied on the use of anthelmintics, and will continue to do so for some time, as no viable alternatives are yet available at the scale needed for effective prevention or control. For human-infective helminths, anthelmintics are distributed primarily via coordinated periodic mass drug administration (MDA) campaigns [5]. For GI helminths of livestock, large-scale and wide-reaching preventative treatment of animals is frequently undertaken; however, the management of anthelmintic use, in terms of drug class [9], dosage and route [10], and timing [11], can vary markedly between or within countries and regions [11–13]. Although anthelmintic treatment is relatively effective, some challenges exist, including: (i) variable efficacy against different worm species and developmental stages; (ii) reinfection soon after treatment [14–16]; and (iii) the evolution of drug resistance [8, 13, 17, 18]. Anthelmintic resistance has evolved as a consequence of the excessive use of single drug classes. In some nematode species of livestock, for example *H. contortus*, resistance is widespread to the point that farming has become unviable in some regions of the world [19, 20]. Although the nature and extent of resistance in human-infective helminths is less understood compared with that in helminths of veterinary significance [21–23], the extensive use of anthelmintics via MDA does put a significant selection pressure on human-infective helminth populations. Thus, the monitoring of drug efficacy should form part of an effective control programme, and improved management strategies need to be implemented to preserve drug efficacy and/or overcome resistance. Although considerable efforts have attempted to develop vaccines against helminths [24–26], no commercial vaccines are in current use against STHs of humans (e.g. [27, 28]), whilst only two vaccines are available for use against GI helminths of livestock, namely, Barbervax® and Wirevax® (Wormvax Australia Pty Ltd.) against *H. contortus* (with short-term protection) in Australia and South Africa, and one vaccine for use against a non-GI helminth, namely, Providean Hidatil EG 95® (Tecnovax) against cystic hydatidosis caused by *Echinococcus granulosus* in South America [28, 29].

Accurate diagnosis remains a critical component of monitoring parasite infections and their response to anthelmintic treatments. The diagnosis of GI helminth infections in humans and live animals usually relies on the microscopic detection/identification of eggs or larvae in faecal samples by faecal smear, faecal flotation and/or larval culture. These methods are used to assess the parasite species present and/or estimate the intensities of infections within individuals, groups or populations [30]. While routinely used, these conventional techniques can be relatively labour intensive and time consuming to perform (unless automated), and their diagnostic performance depends on sample preparation, the reproductive potential of a worm species and/or infection prevalence [31–33]. Molecular methods have been developed to attempt to overcome these limitations [34–39] but, to date, they have been used mainly to address academic research questions and there has been limited uptake in large-scale control programmes. The World Health Organisation (WHO) revised the neglected tropical disease (NTD) roadmap (2021–2030) [40], which sets out a strategic vision to control and ‘eliminate’ 20 NTDs afflicting humans, including STHs. As the control of STHs relies heavily on MDA, consideration must be given to which strategy is best suited to achieving a substantial, measurable and sustained reduction or elimination of transmission, and how novel diagnostics approaches can support sound surveillance strategies [34, 41].

In the present review, we: (i) examine the strengths and weaknesses of conventional methods currently available for the diagnosis of GI helminth infections of humans and animals; (ii) appraise high-throughput molecular barcoding and metagenomic sequencing as tools to determine the taxonomic composition of helminth infections; and (iii) review current knowledge of the interactions between helminths and microbiota in the host gut. The overall aim is to provide a basis to identify biomarkers with the potential to define the health/disease status of individuals and populations, as well as to detect existing or emerging anthelmintic resistance.

## Conventional diagnostic methods

### Coproscopic techniques

Most commonly, microscopy-based methods used to examine faecal samples for helminth eggs include: direct examination of stool smears on a glass slide, such as the Kato Katz technique commonly used to detect STHs and schistosome infections [42]; flotation using a counting chamber (e.g. McMaster), commonly used for detection of infections by GI helminths in livestock; and the morphological identification of larvae following coproculture [43]. Faecal flotation methods often include a centrifugation step to concentrate eggs from samples, such as with the FLOTAC/mini-FLOTAC technique [44, 45]. These methods have been reviewed extensively in previous articles [31, 46, 47].

Although these methods are affordable and widely used around the world, they are not without limitations. The enumeration of eggs per gram (i.e. faecal egg counts [FEC]) is often used to estimate infection intensity, but this relationship is only valid for parasites with high reproductive potential (e.g. hookworms and *Haemonchus* spp.) [46, 48]. This approach is useful in endemic areas and/or settings where the prevalence of infections is high, but evidence indicates that diagnostic sensitivity can be low (43–52% for direct microscopy, depending on STH species) [31], resulting in underestimations of prevalence in field-based studies (cf. [35, 37, 49–52]). Moreover, the specificity of the diagnosis is reliant on the knowledge and skills of the diagnostician, i.e. their ability to conduct the assay in a repeatable/reproducible manner and to accurately identify eggs to genus or species within a relatively short time-frame to overcome challenges linked to egg degradation or hatching. Approaches to automate the analysis of microscopy-based helminth egg detection, including egg concentration through filtration [53], are under development and becoming available, such as the digitized microscopy tool FECPAK^G2^ [54, 55] or Lab-On-A-Disk (LOD), the latter of which combines microfluidics-based separation of helminth eggs [56] with machine-learning for egg identification (cf. *Trichuris* eggs [57]). Whilst the FECPAK^G2^ potentially has a higher throughput than conventional microscopy and does not require a specialist, it necessitates cloud/internet access to make results immediately available [55]. Currently, this tool is used for the diagnosis of livestock helminth infections [55, 58], but the limited sensitivity of FECPAK^G2^–based diagnosis indicates that it will not be suitable for the detection of human STHs in geographical areas with medium or low prevalence of infections [51, 54]. Machine learning-based methods have also been evaluated and applied for the diagnosis of helminthiases (e.g. [59–63]). However, the utility of these recent technological developments for the diagnosis of human helminth infections will depend on their performance in large-scale field surveys and on their cost and benefits [56].

### Coproantigen detection

Immunodiagnostic tools detect parasite antigens in faeces (coproantigens). Enzyme-linked immunosorbent assays (ELISAs) that target parasites affecting companion animals (*Toxocara canis*, *Ancylostoma caninum* and *Trichuris vulpis*) [64], the human STHs *An. ceylanicum* [65] and *A. lumbricoides* [66] and the cestode *Taenia solium* [67] have been developed; although, for the latter, cross-reactivity with other cestode species appears to be an issue [68]. ELISA kits are also commercially available; for example, for the detection of liver fluke (*F. hepatica*) antigens from faeces of both livestock [69] and humans [70]. Results achieved by ELISAs show a strong correlation between coproantigen and copro-DNA levels in stools from individuals with *Ascaris* infection [66], while the correlations between microscopy-based worm burden and real-time (quantitative PCR [qPCR]) are moderate for hookworm [35, 52] or strong for *A. lumbricoides* [35, 52] and *T. trichiura* [52]. It is important to consider that the performance of ELISAs for the detection of coproantigen can be affected by several components in stool samples, including salts, proteases, antibodies and organic compounds, and are dependent on sample preservation and storage; variations in any of these might interfere with antigen detection and lead to false positive or negative results [70]. This also applies to nucleic acid-based methods.

### Nucleic acid detection

Owing to their high analytical sensitivity and specificity [35, 52], nucleic acid amplification techniques (NAATs) are increasingly being used for the copro-diagnosis of helminth infections of humans and animals [34, 36, 71, 72]. NAATs aim to specifically target helminth DNA or RNA in extracts from stool samples via the binding of complementary oligonucleotide primers and subsequent enzymatic amplification of target DNA/RNA. The application of NAATs is important for the detection of helminth infections in low-prevalence settings [34]. Following the WHO’s updated targets for eliminating NTDs (including STHs), qPCR, irrespective of target, is increasingly being recognized as an important diagnostic tool to support both elimination and surveillance efforts [34, 73].

Currently, most of the available PCR-derivative tools target only a small number of helminth species, require suitable laboratory infrastructure (with electricity) and equipment and are usually low throughput, although a few multiplexed attempts offer the advantage of a somewhat higher throughput [38, 74, 75]. These constraints make them less suited to field application settings [76]. To begin to address this issue, recombinase polymerase amplification (RPA) in portable battery-operated instruments or loop-mediated isothermal amplification (LAMP)-based tools have been established for field use. Examples include RPA of *S. haematobium* DNA from genomic DNA from urine [77] and LAMP detection of STHs from nucleic acids extracts from adult worms [78]. To date, most NAATs are based on the amplification of repetitive sequence elements in the genomes of helminths, such as species-specific ribosomal RNA (rRNA) genes [39, 74, 79] or genome-wide, tandem repeats [36, 80].

It appears that high analytical sensitivity and specificity can be achieved using such targets. However, it is not yet known to what extent variation in nucleotide sequence, and/or copy number of repetitive elements, might affect the diagnostic performance of these assays in different geographical regions. This requires critical evaluation.

## Barcoding of helminths by high-throughput sequencing

DNA barcoding is a method for the identification of species using a relatively short DNA region(s) of a gene or genes [81]. For a barcode to be useful, two basic criteria need to be met: (i) the barcode itself must contain sequence variants that differentiate between species, and this variation need to be markedly and consistently higher than levels of variation within individual species, irrespective of the geographical origin of a sample/specimen [82], and (ii) the regions flanking a barcode should be sufficiently conserved between species to allow the design of oligonucleotide primers that consistently bind (irrespective of species) to enable efficient DNA amplification by PCR. Identifying truly universal barcoding targets and primers targeting all helminth species is an ambitious goal yet to be achieved.

For a range of animal groups, DNA barcodes derived from the mitochondrial cytochrome *c* oxidase subunit 1 (*cox*1) gene [83, 84] and nuclear rRNA genes (18S or 28S and variable regions within such genes, such as D4-D6 of 28S rDNA and the V4 or V9 domains of 18S rDNA) are commonly used [85–89]. However, it is often (incorrectly) assumed that DNA barcodes such as those described above are ‘reliable’ or ‘useful’, without prior validation for new groups of organisms. For example, mitochondrial DNA (mtDNA) sequences of parasitic nematodes (including *cox*1) display high levels of synonymous sequence variation within species that limits their utility as barcodes, despite high levels of protein sequence conservation [89, 90]. On the other hand, nuclear rRNA genes (while useful for phylogenetic studies) [91, 92] are usually too conserved in sequence for reliable species identification. The second internal transcribed spacer of nuclear ribosomal DNA (ITS-2) is the only barcode that has been rigorously evaluated as a species marker for selected nematodes (i.e. Strongylida and Ascaridida) [93, 94] as well as for some trematode species [95]; usually, most barcodes have not been critically assessed for widespread application to a broad range of taxonomic groups. The extensive, early taxonomic evaluations of ITS-2 for select nematode groups [94] has paid off, and ITS-2 barcoding by high-throughput sequencing (HTS), enabled by database resources and informatics [96], is now well established for strongylid nematodes to define ‘nemabiomes’ [97–100]. This is not the case for other barcodes, as they do not provide reliable/accurate species markers.

Defining an informative barcode(s) for a wide range of species can be challenging because of a lack of knowledge of the genomes of many species and their taxonomic/phylogenetic relationships. Thus, designing primers to amplify the same barcode from diverse taxa can be problematic [90]. The broader the target group (e.g. family, order and/or phylum), the greater the likelihood that primers designed to the barcode will hybridize and amplify non-specifically in non-target regions of non-target taxa. Thus, it is important that barcodes, associated reagents (particularly primers) and protocols for amplification and sequencing are critically evaluated experimentally prior to being used in large-scale studies, rather than simply relying on barcodes and primers that have been identified or designed in silico alone, as databases do not represent the broader biodiversity [101, 102].

## Metabarcoding to capture helminth diversity

HTS and metabarcoding allow for the targeted amplification of multiple, homologous regions from phylogenetically diverse taxa simultaneously in a mixed sample, and can determine the relative abundance of these regions, as compared to traditional barcoding with its limited capacity to target one species per sample [103, 104]. Conventional barcoding approaches comprise expensive and laborious sequencing steps. However, the marked decrease in cost and increase in accessibility of HTS over the last decade has encouraged novel approaches for the diagnosis or analysis of helminth infections or populations [84, 86–88, 91, 92, 97–100, 105–109]. The sheer volume of sequence output from ‘metabarcoding’ accommodates the use of conserved primers to “capture” and sequence these DNA regions from a diverse range of organisms, with downstream bioinformatics allowing the characterization of species/taxon identity and sequence diversity (both within and among taxa) within a sample. Whilst metabarcoding may provide the relative proportions of species present in a complex sample, currently established pipelines do not yield absolute/measuring values that correspond with the species present. This may be a disadvantage relative to conventional tools, such as microscopy and qPCR, if metabarcoding is proposed to be a diagnostics alternative. In addition, due to its nature of amplifying multiple barcodes simultaneously, it is unclear as to whether the diverse barcodes present in a sample are being amplified and sequenced equally efficiently. Some research groups have accounted for this by including ‘mock parasite populations’ in sequencing runs to normalize read counts and correct species representation bias [97].

A number of target genes have been proposed and tested via metabarcoding approaches to characterize helminths. Based on in silico work, mitochondrial 12S and 16S rRNA genes have been proposed as promising candidates for the metabarcoding of parasitic nematodes [110]; however, one key species of STH, *T. trichiura* (whipworm), was not included in this study. Thus, the utility of 12S/16S rRNA genes as universal barcodes remains to be rigorously assessed and established. Nevertheless, the mainstay of metabarcoding has focused on adapting ribosomal barcoding primers, most commonly ITS-2. A recent comparative analysis of partial mitochondrial 12S and 16S rDNA regions versus partial mitochondrial *cox*1 regions, and of the nuclear genes ITS-1, ITS-2 and 18S rDNA revealed that, although both approaches allow the successful identification of a wide range of nematode species, ITS-2 is best suited to specific identification [110]. Importantly, sequence differences in ITS-2 between species is consistently higher than the low levels of nucleotide variation within species. For these reasons, a reference database of ITS-2 sequences has been established [96] to aid metabarcoding studies of strongylid nematodes.

Metabarcoding using nuclear 18S rDNA and/or ITS-2 has been successfully applied to the specific identification of nematodes from copro-DNA from livestock [97–100] and wildlife animals [86, 107, 111–114] and across a range of roundworm species, including *Ascaridia*, *Heligmosomoides*, *Nippostrongylus* and *Strongyloides* [86]*, Haemonchus, Cooperia* and *Trichostrongylus* [100], and/or cestodes (e.g. *Hymenolepis diminuta*) in wildlife [86] and livestock animals [97–100]. Markers in the 28S rRNA gene have been applied to identify common nematodes of rodents (including *Heligmosomoides*, *Nippostrongylus* and *Strongyloides*) via metabarcoding using faecal DNA [86].

This same concept has been recently expanded to characterize drug-resistant genetic variants [115–117], and offers a promising proof-of-concept to detect new variants for resistance or other genetic markers of traits of interest.

## ‘Shotgun’ metagenomic sequencing

The composition of samples, such as those derived from faeces, can be very complex, containing nucleic acids from a wide range of organisms, spanning species represented in food, the individual’s genome and a wide range of viruses, microbes and pathogens, including parasites [118]. Rather than focusing on a discrete target sequence as described above, shotgun metagenomics aims to use HTS to detect all nucleotide sequences (DNA or RNA) present in a given sample.

Metagenomics tools, in particular when used in epidemiological contexts, might provide additional value over conventional diagnostic approaches. In addition to revealing polyparasitism, such approaches may also reveal genetic variation within and among samples; this information could be of value by inferring the relationships of pathogens/parasites/microbes and can be employed to explore population dynamics over time. Increasingly, metagenomics is being used in applications where the molecular signals for parasites are weak or difficult to resolve. For example, in archaeoparasitology, DNA recovered from ancient settlements, human remains and mummies has been analysed using short genomic regions of up to 400 bp [119, 120], from which mitogenomes have been assembled [121]. Other DNA-based, shotgun metagenomic studies of sediments, combined with gene-specific 18S rRNA gene metabarcoding, revealed *Ascaris* and *Trichuris* spp. eggs present in a cesspit of a nineteenth century palace [119]. Strikingly, whole-genome sequencing analysis has been used to resolve the parasite genomes of preserved *T. trichiura* eggs up to 1000 years old, the oldest eukaryotic pathogens sequenced to date [122].

Although metagenomic-based tools yield substantially more sequence data per sample, a major limitation is that such approaches are relatively more expensive, both in terms of infrastructure, equipment and cost per sample, even when a pooling strategy is applied. Furthermore, high technical skills (i.e. for bioinformatics processing of sequence data) and computational capacity (e.g. cloud-based or physical computational capacity for performing analysis) are usually required, both of which require extensive expertise and further expense. In the short term, this may require outsourcing to centralized facilities or commercial companies; however, capacity is growing throughout the world, particularly in hubs in low- to middle- income countries and will likely enable the development of suitable technologies and expertise in endemic countries over time. One significant advantage is that, once established, these technologies (and particularly metabarcoding) have the capacity to scale to very high throughput, which will help markedly reduce the costs per sample. Further cost reductions could be achieved by performing low coverage “genome skimming”, which specifically targets high copy number regions of genomes, such as repetitive elements in nuclear DNA and in mitochondrial DNAs [123], which could allow metagenomics to be performed at scale. Examples of the application of metagenomics or metabarcoding for the identification of helminth nucleic acids in faecal extracts and/or faecal cultures from humans and animal species are summarized in Table 1.

**Table 1:** Examples of metabarcoding and metagenomics approaches for the detection of helminth species in faecal samples or cultures

Methodology	Phylum	Nucleic acid of choice	Host	Platform	Matrix	Gene/marker or reference loci	References
NA	Nematoda	DNA	NA	NA	In silico	12S, 16S, ITS-2	[110]
Metabarcoding	Nematoda	DNA	Rat	MiSeq Illumina	Faeces	18S (V4-V5, V7-V8, V9)	[86]
Metabarcoding	Nematoda	DNA	Rat	MiSeq Illumina	Faeces	28S (D3-D4, D4-D5, D3-D5, D4-D6)	[86]
Metabarcoding	Platyhelminthes	DNA	Rat	MiSeq Illumina	Faeces	18S (V4-V5, V7-V8, V9)	[86]
Metabarcoding	Platyhelminthes	DNA	Rat	MiSeq Illumina	Faeces	28S (D3-D4, D4-D5, D3-D5, D4-D6)	[86]
Metabarcoding	Platyhelminthes	DNA	Various	NA	Environmental	18S (V4, V7,V8-V9, V9)	[87]
Metabarcoding	Nematoda	DNA	Rat	MiSeq Illumina	Worms	18S	[88]
Metabarcoding	Cestoda	DNA	Rat	MiSeq Illumina	Worms	18S	[88]
Metabarcoding	Nematoda	DNA	Cattle	MiSeq Illumina	Larvae/faeces	ITS-2	[97]
Metabarcoding	Nematoda	DNA	Sheep	MiSeq Illumina	Larvae	ITS-2	[98]
Metabarcoding	Nematoda	DNA	Cattle	MiSeq Illumina	Larvae	ITS-2	[99]
Metabarcoding	Nematoda	DNA	Sheep	MiSeq Illumina	Eggs and larvae	ITS-2	[100]
Metabarcoding	Nematoda	DNA	Rufus mouse lemur	454 Roche	Larvae	18S	[107]
Metabarcoding	Nematoda	DNA	Wild reindeer	MiSeq Illumina	Larvae	ITS-2	[111]
Metabarcoding	Nematoda	DNA	Sheep	MiSeq Illumina	Larvae	ITS-2; see also [97]	[116]
Metabarcoding	Nematoda	DNA	Roe deer	MiSeq Illumina	Larvae	ITS-2; see also [97]	[113]
Metabarcoding	Nematoda	DNA	Wild ruminants	MiSeq Illumina	Larvae	ITS-2; see also [97]	[112]
Metabarcoding	Nematoda	DNA	Bison	MiSeq Illumina	Larvae	ITS-2; see also [97]	[108]
Metabarcoding	Nematoda	DNA	Horse	MiSeq Illumina	Larvae	ITS-2; see also [97]	[109]
Metabarcoding	Nematoda	DNA	Gorillas, Mangabey	MiSeq Illumina	Larvae	ITS-2	[114]
Metagenomics	Nematoda	DNA	Primate	Hiseq Illumina	Faeces	18S, COI	[105]
Metagenomics	Nematoda, Platyhelminthes	DNA	Human	MiSeq Illumina	Faeces	Unavailable	[106]

*ITS-2* Second internal transcribed spacer, *NA* not applicable

In instances where shotgun metagenomics approaches are used for the genetic identification and characterization of parasites, the number of sequence reads derived from the parasites within a given dataset might be substantially smaller than those originating from host and microbiome within the gut. Dependent on the aim and application, parasite-derived DNA and sequencing reads may be enriched by: (i) filtering the sample to collect worm eggs prior to DNA extraction; (ii) targeting parasite DNA via selective/whole genome amplification [124]; and/or (iii) bioinformatic selection of parasite sequences prior to downstream analyses (via, for instance, mapping to reference genomes when available). Recent genomic investigations, databases and resources underpin metagenomic investigations. In particular, the 50 Helminth Genomes Project [125] has generated extensive genomic resources for many important helminths of human and veterinary importance, and open-access data repositories, such as WormBase ParaSite [126]. The Earth BioGenome Project [127], which aims to sequence all eukaryotic life on earth, also promises to improve resources for helminths to support metagenomic analyses in the future.

One important feature of sequence data generated using shotgun metagenomic approaches is that it may provide end-users with the opportunity to not only identify known or possible pathogens within faecal samples, but also to simultaneously genetically characterize the microbial communities from within the GI tract of the vertebrate host and to explore interactions among parasites, microbes and the host.

## Metabarcoding and/or metagenomics to define microbial signatures that associate with helminth-infections

Over the last decade, evidence has emerged that infections by soil-transmitted and GI helminths are accompanied by profound qualitative and quantitative modifications in the composition of the faecal microbiome of their hosts, although, to date, studies have mainly focused on the host bacteriome [128–130], rather than the virome [131] or mycobiome [132]. Modifications of the bacterial composition of the host microbiome can be characterized using microbiota-targeting metabarcoding and/or metagenomic approaches. Metabarcoding sequencing of one or more hypervariable regions of the bacterial 16S rRNA gene has been applied often to characterize faecal microbial profiles of both humans and animals infected by parasitic helminths [129, 133–135], both under experimental [136–138] and natural conditions of infection [139–143], and before and after anthelmintic treatment [143, 144]. Examples include the characterization of the faecal microbiota of cohorts of human volunteers with coeliac disease or multiple sclerosis experimentally infected with *N. americanus* [137, 145], and individuals naturally infected by STHs [142–146]. In relation to nematode species of veterinary importance, metabarcoding, using the bacterial 16S rRNA gene, has been applied to, for example, the characterization of the faecal microbiota of *H. contortus*-infected goats [147]*, T. circumcincta*-infected sheep [148], or *Ostertagia ostertagi-*infected cattle [149].

Information on specific qualitative and/or quantitative alterations of microbial taxa in faecal samples from helminth-infected humans or animals have been reviewed elsewhere [128–130]. Often, direct comparisons of faecal microbial profiles of helminth-infected humans and/or animals compared with corresponding uninfected hosts lead to contrasting results (reviewed in [129]), even between data sets generated from the same host–parasite pair under similar experimental conditions [148]. Such discrepancies are likely linked to variability in structure and composition of the vertebrate gut microbiota [130, 134, 150]. Furthermore, intrinsic and/or extrinsic factors, including, but not limited to, age, gender, diet and/or underlying health conditions, all exert profound effects on the composition of the vertebrate gut microbiota [128, 129, 143]. Such inter-individual variability makes the discovery of reliable microbial-based biomarkers of helminth infection a challenging task; nevertheless, in a recent study, specific biomarker-discovery algorithms were applied to the identification of unique populations of faecal bacteria associated with worm colonization in *T. circumcincta*-infected sheep in an experimental system [148]. Whilst this study represents a promising step forward in the discovery of microbiota/microbiome-targeting biomarkers of helminth infections in both humans and animals, individual investigations examining the impact of worm colonization on gut microbial communities are often compromised by insufficient sample size and, thus, low statistical power [129]. A possible solution is to combine available datasets generated from a wide range of host–parasite pairs [151] and, in turn, the number of faecal samples needed to achieve sufficient statistical power to underpin robust biomarker discovery studies. Understanding the interaction between helminth infections and the host microbiome could unveil microbial signatures associated with such infection(s) (or not). Some recent studies indicate that species-specific microbial signatures are associated with helminth infections and worm burden [142], whilst a study in Cameroon showed how bacterial signatures can predict polyparasitism with 81–82% accuracy for *A. lumbricoides* or *T. trichiura* [152]. The same study demonstrated how diet and lifestyle are associated with different microbial signatures. In turn, selected microbiome populations could confer resistance or susceptibility to infection [141, 146, 150], or even act as a means of prophylaxis and treatment (e.g. by means of probiotics). Clearly, faecal sample extracts are ideal specimens for non-invasive biomarker-based diagnosis of helminth infections, as they contain end-products of host, parasite and/or microbiome metabolisms, and/or of the crosstalk between them; these products could represent an untapped source of potential diagnostic markers.

## The prospect of metabolites as biomarkers

In addition to nucleic acids, vertebrate faeces contain a vast array of molecules originating from metabolic and catabolic processes from the host, the gut microbiome and the helminths themselves. Such molecules, generated by individual organisms or through the interactions between organisms, represent a potential source of novel biomarkers of infection(s).

Metabolomics aims to detect and characterize small molecules (e.g. amino acids, peptides, carbohydrates and fatty acids) involved in metabolic pathways in a single biological specimen. This discipline takes advantage of a plethora of ‘omics’ tools (e.g. chemogenomics) and platforms (liquid/gas chromatography coupled with mass spectrometry) to facilitate metabolite discovery and characterization [153–155]. Thus far, application of metabolomics techniques to the diagnosis of infectious diseases from faecal extracts remains limited. One example has demonstrated that symptomatic infection by the bacterium *Clostridioides difficile* was correlated with high levels of 4-methylpentanoic acid, a product of Stickland metabolism, in faecal extracts [156].

Excretory/secretory products (ESPs) from parasitic helminths are also known to contain a large number of metabolites that might serve as useful diagnostic biomarkers. For example, a recent study aimed at characterizing the metabolic “footprint” of *Echinococcus multilocularis* revealed metabolites (e.g. acetate and alanine) secreted or excreted by the parasite in vitro that could be further explored as diagnostic target candidates [157]. However, their specificity remains to be established, as the same panel of metabolic markers may be excreted/secreted by more than one parasite. Indeed, the same metabolites (e.g. myristic acid, lauric acid) were detected in ESPs from *T. muris*-infected mice [158], as well as from dogs infected by *A. caninum* [159]. Based on this observation, these metabolites are likely to be unsuitable as species-specific diagnostic targets for helminth infections. In the same study [158], more than 30 other metabolites were identified in ESPs isolated from *N. braziliensis*-infected rats or *T. muris-*infected mice, but, again, the suitability of these metabolites as potential biomarkers of infection remains to be established. In contrast, ESPs isolated from the dog roundworm *T. canis* were enriched for talose, an uncommon carbohydrate, which may represent a biomarker [160].

Changes in the composition and function of the host gut microbiome can be exploited in studies aimed at discovering novel infection biomarkers in helminth-infected subjects. In a recent study, Jenkins et al. [136] conducted a metabolomics analysis of faecal samples from a cohort of human volunteers pre-diagnosed with *S. stercoralis* infection, and compared data with that obtained from a cohort of uninfected individuals from the same geographical area. In this study, faecal extracts from *S. stercoralis*-infected subjects displayed an increased abundance of selected amino acids (leucine, alanine and lysine) compared with uninfected individuals. Whilst the number of subjects enrolled in this study was limited, and targeted metabolomics techniques (in lieu of broad-spectrum techniques) were applied to the characterization of the set of metabolites in faecal extracts, data from this study appear to provide a sound starting point for the exploration of bacterial secondary metabolites as bystander signatures of helminth infection. In turn, the characterization of worm-associated changes in bacterial and/or host metabolism might lead to a better understanding of the pathophysiology of helminth infections, and thus to the discovery and development of novel and improved treatment strategies aimed at assisting the restoration of gut homeostasis. Whilst faecal metabolites currently represent an untapped source of potential biomarkers for helminth infections, other molecules, such as non-coding short RNAs (e.g. microRNAs) of worms show promise, as reported in recent serological studies of *S. mansoni* in humans from endemic areas, as well as in rats experimentally infected with *S. japonicum* [161].

## Conclusions

Stool can be seen as a window into a complex ecosystem in the GI tract of animals. Infectious agents detectable in stool encompass eukaryotes (including parasitic helminths, protists and fungi), bacteria, viruses and archaea. Thus, stool is a critically important biological matrix to reveal the composition of, and interactions between, organisms colonizing the GI tract of both healthy and diseased individuals.

**Fig. 1:**
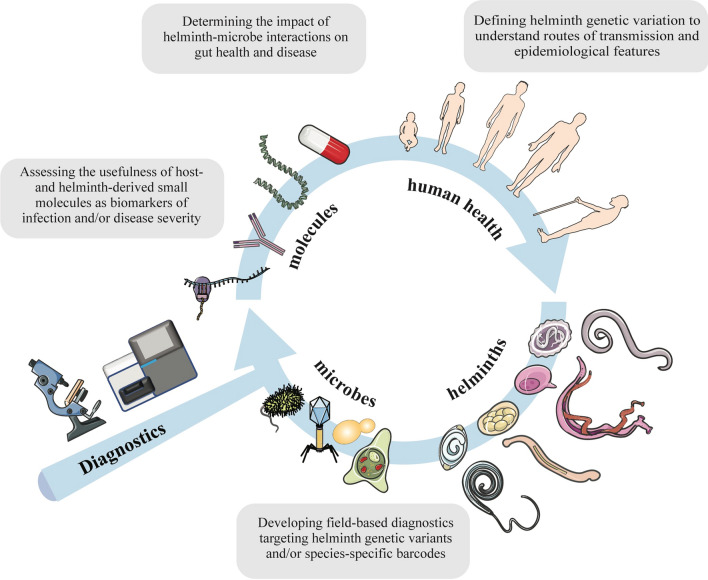
A proposed systems-biology approach for diagnosing and characterizing signatures of worm infection in stool. The gut is a complex environment hosting a plethora of micro- and macro-organisms. The application of metabarcoding and metagenomics-metabolomics tools to the identification and characterization of parasite populations (including their genetic variants), as well as of host- and gut microbiome by-products of worm colonization will offer unique opportunities to: (i) better define the diversity of helminth populations; (ii) discover and develop sensitive and specific diagnostic tools; and (iii) dissect the complex relationships between gut function and pathophysiology of helminth disease. Figure elements used license-free courtesy smart.servier.com.

From conventional molecular methods to more advanced methods of shotgun or deep amplicon sequencing, the field of helminthology makes use of a diverse array of tools for both the detection, identification and characterization of helminths in faeces and other matrices. However, the translation of these tools to the field of diagnostics operated by health workers and programme managers is challenging, particularly in low- and middle-income countries, and, thus far, has been mainly limited to veterinary applications. Although currently available in an academic research context, metabarcoding and metagenomics have a notable capacity to increase the scale and throughput in analytical and diagnostic investigations, and have major potential to enhance our knowledge and understanding of parasite–microbe interactions and relationships and of species diversity within host individuals and populations, and/or guide chemotherapeutic and parasite control. Such a systems-biology approach (cf. Fig. 1) might also be used to facilitate the monitoring of the dynamics of worm populations and the success of MDA programmes. Clearly, well-controlled studies are needed in the future to establish novel microbiome- or host-specific signatures that correlate with worm infections. As the field of metabolomics grows, curated databases containing published findings and data sets are necessary to facilitate the direct comparison of metabolic profiles and define useful biomarkers or signatures (cf. [154]). Coordinated efforts in this area to improve diagnostic and analytical tools could transform our knowledge of parasite-microbe interactions and contribute to better surveillance, treatment and control of GI helminths of humans and animals.

## Data Availability

Not applicable.
